# Coronary septic embolism presenting as acute myocardial infarction. A case report of a singular manifestation of infective endocarditis

**DOI:** 10.1093/ehjcr/ytaf037

**Published:** 2025-01-29

**Authors:** Mariana Ferreira Carvalho, Carolina Gonçalves, Beatriz Saldanha Santos, João Morais

**Affiliations:** Cardiology Division, Unidade Local de Saúde da Região de Leiria, Rua das Olhalvas, Pousos, 2410-197 Leiria, Portugal; Cardiology Division, Unidade Local de Saúde da Região de Leiria, Rua das Olhalvas, Pousos, 2410-197 Leiria, Portugal; Cardiology Division, Unidade Local de Saúde de Coimbra, Praceta Professor Mota Pinto, Celas, 3004-561 Coimbra, Portugal; Cardiology Division, Unidade Local de Saúde da Região de Leiria, Rua das Olhalvas, Pousos, 2410-197 Leiria, Portugal; Center for Innovative Care and Health Technology, Leiria Polytechnique, Praceta Professor Mota Pinto, Celas, 3004-561 Coimbra, Portugal

**Keywords:** Infective endocarditis, Acute coronary syndrome, Myocardial infarction, Septic embolism, Case report

## Abstract

**Background:**

Acute ST-elevation myocardial infarction (STEMI) complicated by infective endocarditis (IE) presents a unique challenge in clinical management, especially when associated with septic embolism leading to coronary artery occlusion.

**Case summary:**

The current clinical report describes the case of a 72-year-old male with a history of arterial hypertension, dyslipidaemia, and severe obstructive sleep apnea. The patient presented with anterior STEMI due to an embolic occlusion in the left anterior descending (LAD) artery, secondary to IE. Coronary angiography revealed embolic occlusion at the LAD origin, and balloon angioplasty without stent placement was performed, considering the embolic and infectious nature of the occlusion. Despite targeted interventions, including broad-spectrum antibiotics and support for cardiogenic shock, the patient's condition deteriorated, leading to cardiac arrest and subsequent death on the fourth day of hospitalization.

**Conclusion:**

This case emphasizes the critical need for adapting STEMI management in the presence of IE. It highlights the importance of considering IE in STEMI differential diagnosis and adjusting intervention strategies accordingly.

**Learning points**

- Infective endocarditis (IE) should be considered in the differential diagnosis of STEMI, particularly when cardiogenic shock is present.
- Coronary embolism, though rare, is a potential cause of acute myocardial infarction and requires a tailored approach to intervention, such as balloon angioplasty without stent placement to avoid further complications.
- Early echocardiography is critical in identifying underlying conditions like IE in patients presenting with acute myocardial infarction and hemodynamic instability.

## Introduction

Acute ST-elevation myocardial infarction (STEMI) and cardiogenic shock are critical emergencies in cardiovascular medicine, leading to high mortality rates⁠⁠⁠⁠.^1^

While the main causes of STEMI are well-documented, including plaque rupture or plaque erosion and thrombosis⁠,⁠⁠⁠^2^ rare instances occur where emboli from infective endocarditis (IE) vegetations precipitate these conditions⁠⁠⁠. IE is an infection of the endocardial surface of the heart typically affects the heart valves and can lead to the formation of vegetations⁠. These vegetations may embolize and occlude coronary arteries, causing STEMI.^3^ This case report highlights a unique presentation of STEMI and subsequent cardiogenic shock triggered by an embolus from a vegetation on the aortic valve secondary to IE, elucidating the diagnostic challenges, management complexities, and the crucial role of timely intervention. It underlines the importance of considering IE in the differential diagnosis of STEMI, especially in patients presenting with cardiogenic shock.

## Summary figure

**Figure:**
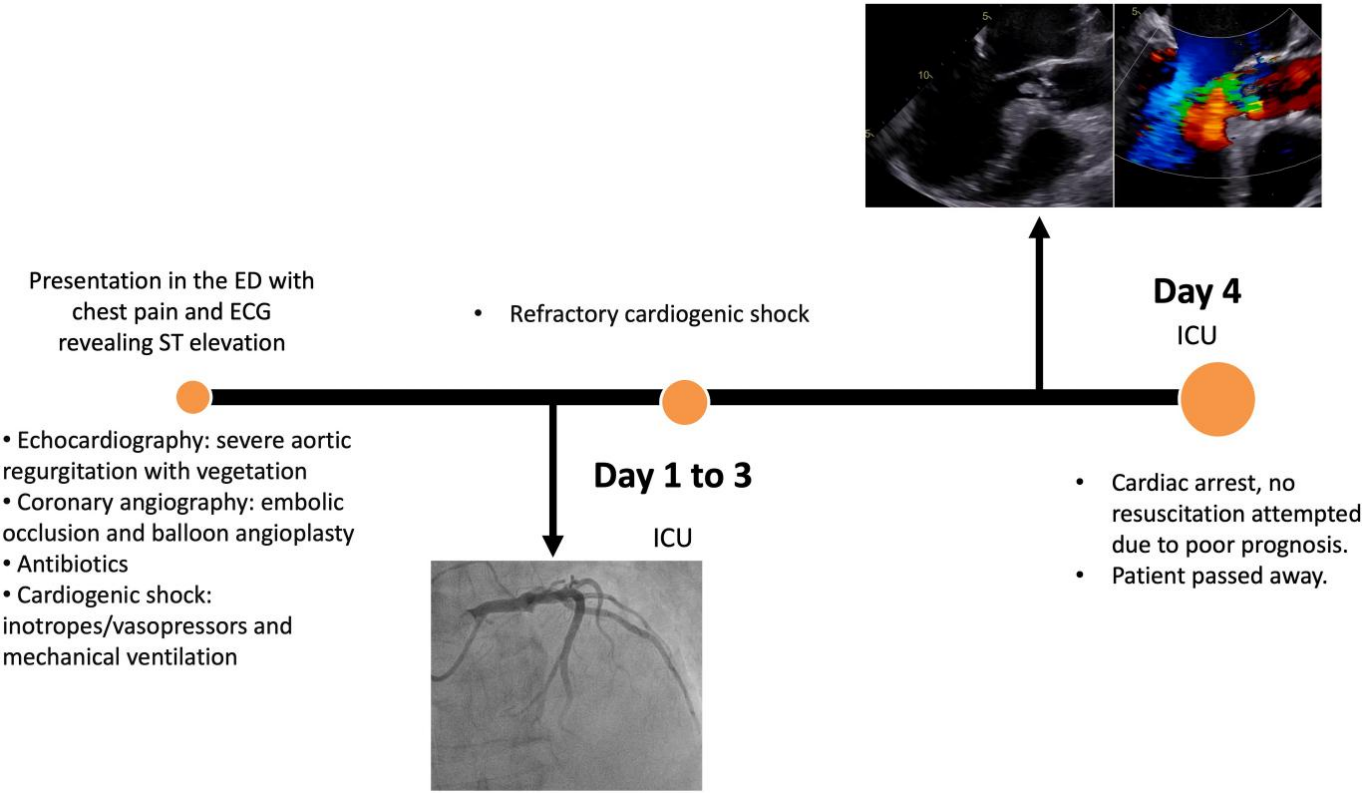


## Case presentation

The patient was a 72-year-old male with a medical history including arterial hypertension, dyslipidaemia, severe obstructive sleep apnoea managed with continuous positive airway pressure, prostate cancer treated with radiotherapy and hormone therapy, and chronic alcohol consumption (∼1 L of wine per day). He worked in the construction and railway industries and owned domestic animals. He denied any family history of cardiovascular disease or history of illicit drug use, as confirmed during the initial clinical assessment. His prescribed medications included atorvastatin 20 mg for dyslipidaemia, venlafaxine for depression, Maltofer 357 mg/5 mL for iron deficiency, and alprazolam 0.5 mg daily for anxiety management.

The patient was brought in by emergency medical services after experiencing intense, crushing chest pain that awoke him from sleep the night before presentation. The pain, described as a heavy weight on his chest with radiation to the right scapula, was accompanied by profuse sweating and acute onset of breathlessness in the early morning hours.

Upon arrival, the patient appeared diaphoretic and pale, with evident signs of poor peripheral perfusion. Vital signs revealed high blood pressure (148/100 mmHg), sinus tachycardia (150 b.p.m.), and hypoxemia (O_2_ sat 88% on 4 L of O_2_). Cardiovascular examination noted a rapid, rhythmic heart rate with aortic diastolic murmur, and pulmonary auscultation revealed bilateral crackles. His abdomen was soft, non-tender, and the extremities were cold, suggesting systemic hypoperfusion.

Initial laboratory results showed elevated high-sensitivity cardiac troponin T (0.32 ng/mL), creatinine (1.8 mg/dL), elevated liver enzymes (AST 56 U/L, ALT 60 U/L), and markers of infection (CRP 120 mg/L, WBC 15 000/mm³). Blood cultures grew *Streptococcus mitis*.

**Figure 1:**
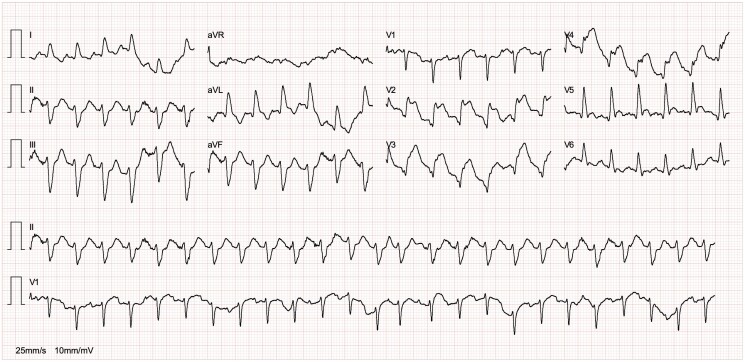
Twelve-lead electrocardiogram at admission showing sinus tachycardia with ST-segment elevations in leads V2-V5 indicating an anterior ST-elevation myocardial infarction (STEMI).

The initial ECG showed sinus tachycardia with ST-segment elevations in leads V2-V5, indicating an anterior STEMI (*Figure 1*). Bedside echocardiography and transoesophageal echocardiography (TEE) revealed left ventricular enlargement, reduced systolic function, with large mobile vegetation on the aortic valve (*Figure 1*) leading to severe aortic regurgitation characterized by a central jet width >65% of the left ventricular outflow tract, vena contracta width of 6 mm, holodiastolic flow reversal in the descending aorta, and slight pericardial effusion, compatible with IE (see Supplementary material online, *Videos S1* and *S2*). Immediate management included intravenous administration of furosemide, nitrates, morphine, heparin, and loading doses of aspirin and ticagrelor. Emergent coronary angiography identified an embolic occlusion at the origin of the left anterior descending (LAD) artery, confirming the embolic source of the STEMI. Despite only achieving a TIMI flow grade of 2 following balloon angioplasty, this outcome was considered acceptable given the patient's IE and the embolic nature of the occlusion. The decision to perform balloon angioplasty without stent placement was strategic, aiming to minimize the risk of dislodging or spreading the infectious material further within the coronary system. IE was thus confirmed after TEE, based on the modified Duke's criteria, due to echocardiographic findings and positive blood cultures.

Recognizing the severity of his clinical status, the cardiac surgery team was consulted early during hospitalization. However, they deemed the patient inoperable due to the high surgical risk and the advanced stage of the disease. Key considerations included refractory cardiogenic shock with multi-organ failure, persistent sepsis despite broad-spectrum antibiotics, and severe aortic regurgitation contributing to haemodynamic instability. Additionally, his critical clinical condition precluded safe transfer to another centre, further elevating the operative mortality risk.

Despite intensive care measures, including mechanical ventilation, vasopressor and inotropic support with high-dose noradrenaline and dobutamine, his condition continued to deteriorate. The patient experienced refractory cardiogenic shock. He was not a candidate for Impella due to severe aortic regurgitation and could not receive extracorporeal membrane oxygenation because this device was unavailable at our centre. The clinical picture suggested that cardiac arrest resulted from a combination of persistent cardiogenic shock, worsening sepsis, and multi-organ failure, culminating in death on day four despite maximal supportive measures.

## Discussion

Coronary embolism, although uncommon, should be considered as a potential cause of acute myocardial infarction (AMI) in patients with conditions such as atrial fibrillation, prosthetic heart valves, and IE. In such scenarios, thrombi or vegetations might embolize to the coronary arteries.^4^ While systemic embolism occurs in about half of IE cases, coronary embolism is less common, with an incidence of approximately 2–3%.^5^ The likelihood of embolism increases when vegetations exceed 1 cm in size, exhibit motility, and are associated with a C-reactive protein level above 40 mg/L, as observed in the patient discussed.^6^

The coexistence of STEMI and aortic valve IE highlights the complexity of managing acute cardiac events. Cardiogenic shock required a prompt, integrated approach extending beyond standard myocardial infarction treatment. Emergent echocardiography in the emergency department was fundamental to diagnosing IE in the setting of AMI, aligning with guidelines indicating that all patients presenting with cardiogenic shock or hemodynamic instability should undergo emergency TTE to try to identify the underlying cause.^7^

Conversely, current guidelines advocate for an early invasive strategy in STEMI management, which was adhered to in this case with immediate coronary angiography. However, the discovery of aortic valve vegetations led to a strategic deviation from typical percutaneous STEMI management, opting for balloon angioplasty without stent placement to minimize the potential consequences of further embolization, mycotic aneurysm formation, or stent infection,^8^ even though there are no clear guidelines addressing this specific medical emergency. This approach emphasizes customized patient care, carefully balancing the benefits and risks of invasive procedures, especially in cases complicated by IE.

Moreover, the role of P2Y12 inhibitors as pre-treatment in STEMI also presents an important consideration. Although there is a biological rationale for early use of these drugs, recent updates in the European Society of Cardiology guidelines for acute coronary syndromes have downgraded the recommendation of routine pre-treatment with P2Y12, reflecting the lack of significant evidence supporting routine use in all cases of STEMI.^7^ This consideration was particularly pertinent in this case, where stenting was forgone after identifying that myocardial injury was caused by septic emboli rather than atherosclerotic coronary plaque rupture. In this context, the premature administration of P2Y12 inhibitors unnecessarily increased the bleeding risk, complicating the decision-making process around potential surgical interventions, which were eventually deemed excessively hazardous.

This case highlights the exceptional rarity of coronary septic embolism as a cause of STEMI, occurring in less than 1% of IE patients. Clinicians must consider non-atherosclerotic causes in atypical STEMI presentations. Diagnostic challenges are significant, requiring comprehensive evaluations including TEE, TTE, CT-PET, and microbiological tests to confirm IE and identify the embolic source. Management of STEMI in this context is complex. The decision to perform balloon angioplasty without stent placement minimizes the risks of further embolization and infection, as stents can become sites for infection and thrombus formation in these patients. Additionally, this case emphasizes reconsidering the use of P2Y12 inhibitors as pre-treatment in STEMI, particularly when myocardial injury results from septic emboli rather than atherosclerotic plaque rupture, due to increased bleeding risks. This underscores the need to update guidelines for managing such complex cases in clinical practice.

## Supplementary Material

ytaf037_Supplementary_Data

## Data Availability

The data underlying this article will be shared upon reasonable request to the corresponding author.

## Lead author biography

Mariana Ferreira Carvalho, MD, is a Cardiology Fellow physician at Unidade Local de Saúde da Região de Leiria EPE, Portugal. She plans to pursue a career in interventional cardiology. Her research interests include acute coronary syndromes, percutaneous coronary intervention and heart failure.
